# Supplementary material for: Preparation of Low-Molecular-Weight Fucoidan with Anticoagulant Activity by Photocatalytic Degradation Method
Source: Foods. 2022 Mar 13;11(6):822. doi: 10.3390/foods11060822 (PMC8954839; doi:10.3390/foods11060822)
Supplement: Supplementary file 1 [file foods-11-00822-s001.zip › foods-1613195-supplementary.pdf]

Supplementary materials

Supplemental Methods

1 SEM observation

2 TEM observation

3 Determination of sulfate group

4 Determination of sulfated polysaccharide

Supplemental Table

Supplemental Table S1. Relative contents of different oligosaccharides produced by photocatalytic degradation

Supplemental Figures

Supplemental Figure S1. SEM images of TiO_2. 100,000× TiO_2 (a); 50,000 × TiO_2 (b); 30,000 × TiO_2 (c); 10,000 × TiO_2 (d).

Supplemental Figure S2. TEM images of TiO_2. 40,000× TiO_2 (a); 80,000 × TiO_2 (b); 150,000 × TiO_2 (c); 400,000 × TiO_2 (d).

Supplemental Figure S3. Molecular weight distribution of fucoidan degraded with 1% TiO_2, 0.95% H_2O_2 in the absence of light.

Supplemental Figure S4.MS^2 of PMP-labeled oligosaccharides from the DF-3.

Supplemental Figure S5. The pseudo-molecular ions of PMP-labelled oligosaccharides from the DF-3.

Supplemental Methods

1. SEM observation

The surface morphologies of Titanium dioxide (TiO_2) were observed by JSM-7800F scanning electron microscope (Japan Electron Optics Laboratory, Tokyo, Japan). The lyophilized powders of samples were sprayed with a thin gold layer, and fixed onto a copper stub, respectively. Then they were observed under the condition of high vacuum at the voltage of 3.0 kV.

2. TEM observation

The micromorphology of TiO_2 was observed using a JEM-2100 electron microscope (JEOL, Tokyo, Japan). The TiO_2 suspension (1 mg/mL) dip-coated on a 300-mesh copper grid coated with a carbon film was air-dried at room temperature before transmission electron microscopy (TEM).

3. Determination of sulfate group

The sulfate group content was determined by the $BaCl_2$-gelatin turbidity method, using K_2SO_4 as the standard. The content was calculated according to the following formula: Sulfate group content (%) = $(\frac{f \times C \times d}{W}) \times 100$, where in this formula, f stands for the conversion factor, C is the determined value of the sample, d is dilution ratio and W is the mass of fucoidan sample. And f should be calculated according to the following formula: f = w/cd (w: the mass of SO_4^{2-}, c: the measured mass of SO_4^{2-}, d: dilution ratio)

4. Determination of sulfated polysaccharide

The content of sulfated polysaccharide was determined by the metachromatic assay with 1,9-dimethylmethylene blue (DMB) at 525 nm by UV-visible spectrophotometer, using fucoidan as the standard. A standard curve was calculated and the corresponding content of standard deduced from the regression equation.

$$\text{Sulfated polysaccharide content (\%)} = \frac{M_{measured}}{M_{sample}} \times 100$$

Supplemental Table

Table S1. Relative contents of different oligosac-charides produced by photocatalytic degradation.

Peak	Ratio	Peak	Ratio	Peak	Ratio	Peak	Ratio	Peak	Ratio
1	3.4	6	4.2	11	7.8	16	3.9	21	6.5
2	4.3	7	3.0	12	2.8	17	3.8	22	12.7
3	2.4	8	1.0	13	3.1	18	8.8	23	8.4
4	1.7	9	7.0	14	1.8	19	6.0	24	6.5
5	3.8	10	4.1	15	2.5	20	10.4	25	5.9

Supplemental Figures

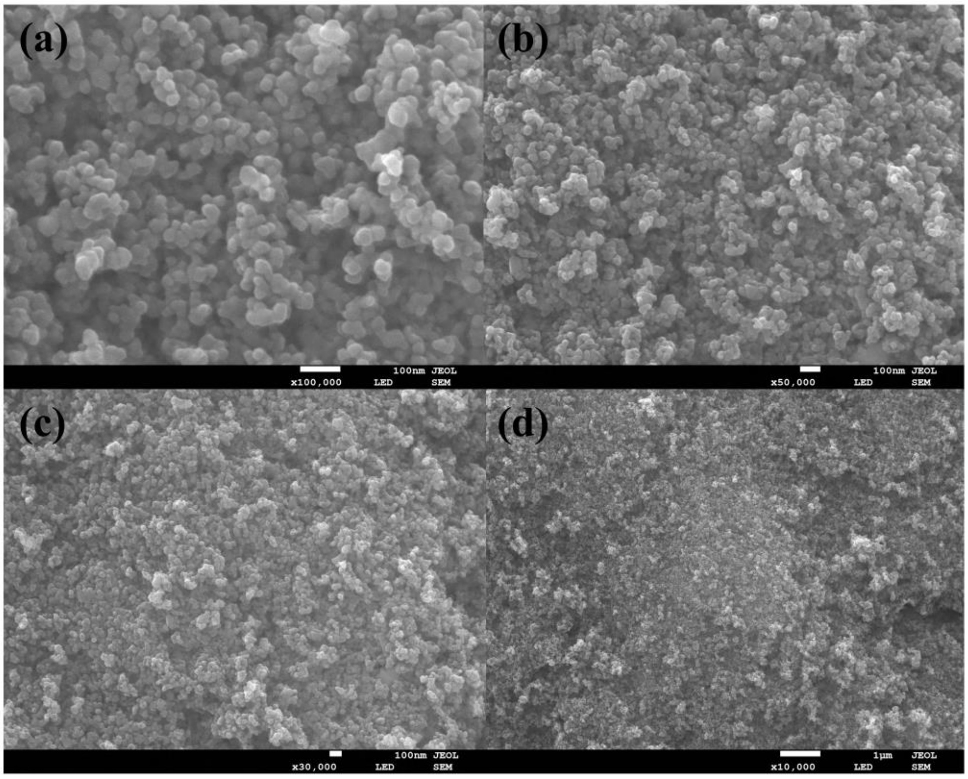

Figure S1. SEM images of TiO_2. 100,000× TiO_2 (**a**); 50,000 × TiO_2 (**b**); 30,000 × TiO_2 (**c**); 10,000 × TiO_2 (**d**).

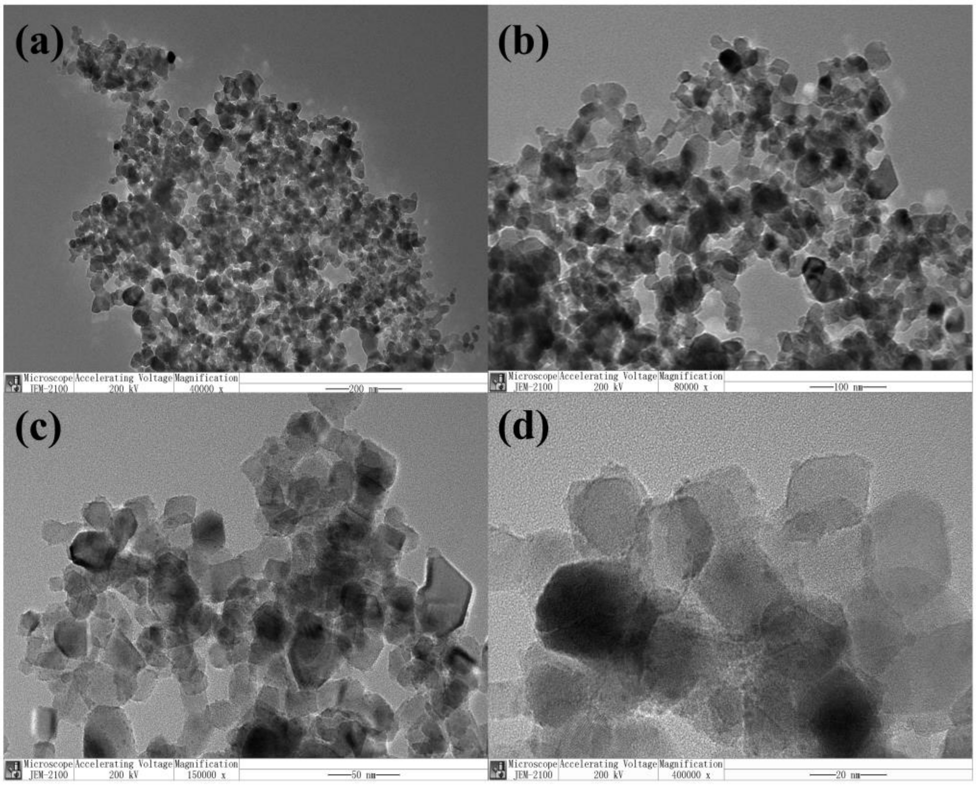

Figure S2. TEM images of TiO_2. 40,000× TiO_2 (**a**); 80,000 × TiO_2 (**b**); 150,000 × TiO_2 (**c**); 400,000 × TiO_2 (**d**).

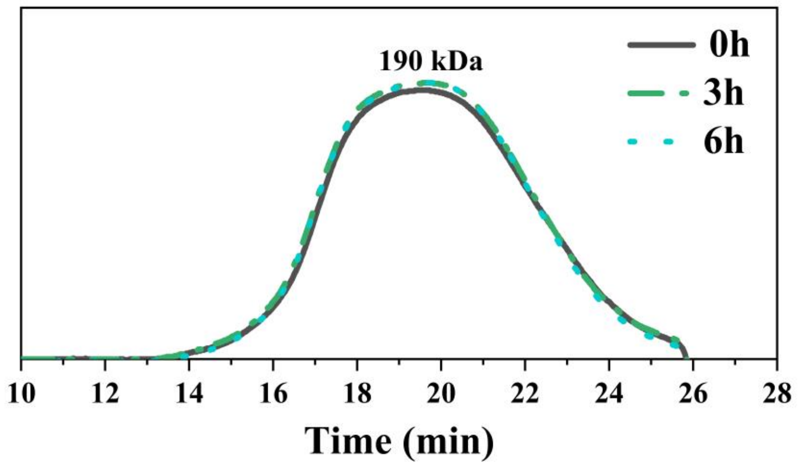

Figure S3. Molecular weight distribution of fucoidan degraded with 1% TiO_2, 0.95% H_2O_2 in the absence of light.

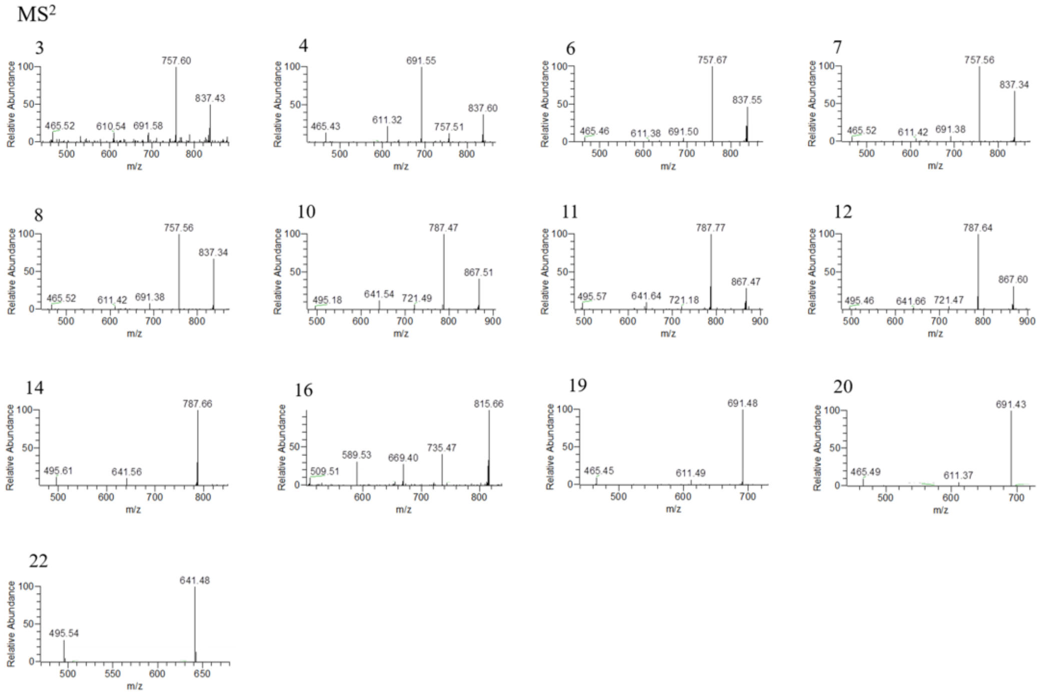

Figure S4. MS^2 of PMP-labeled oligosaccharides from the DF-3.

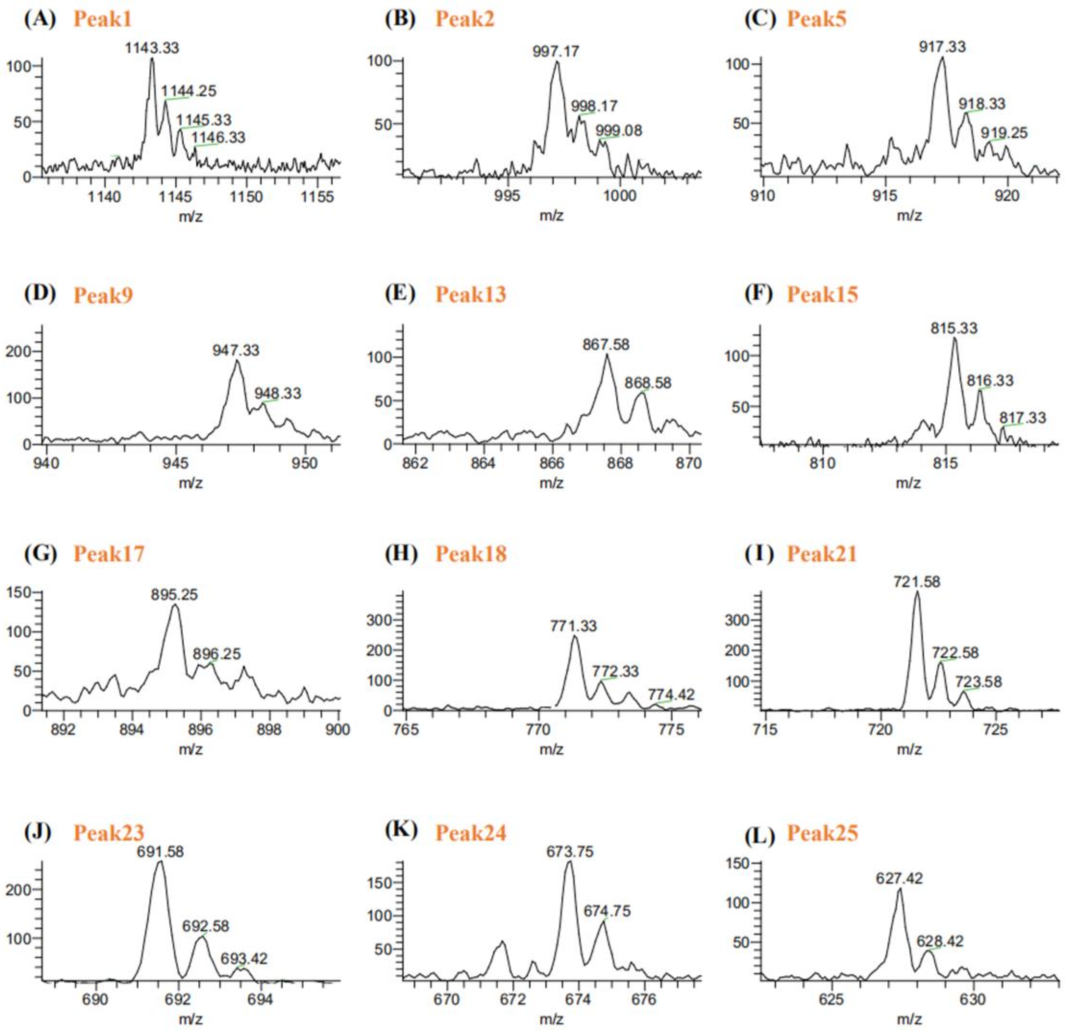

Figure S5. The pseudo-molecular ions of PMP-labelled oligosaccharides from the DF-3.
